# Supplementary material for: Effect of Antibiotic Exposure on Staphylococcus epidermidis Responsible for Catheter-Related Bacteremia
Source: Int J Mol Sci. 2023 Jan 12;24(2):1547. doi: 10.3390/ijms24021547 (PMC9863639; doi:10.3390/ijms24021547)
Supplement: Supplementary file 1 [file ijms-24-01547-s001.zip › ijms-2017192-supplementary.pdf]

Supplementary data

Table S1. Site of isolation and antibiotic resistance profiles of the 36 *S. epidermidis* isolated from catheter-related bacteremia.

Data		N (%)
**Site of isolation**	PICC line	12 (33%)
	Central Veinous Catheter (jugular)	6 (16.7%)
	Implanted access port	6 (16.7%)
	Central Veinous Catheter (subclavian)	4 (11.1%)
	Central Veinous Catheter (femoral)	3 (8.3%)
	Umbilical venous catheter	1 (2.8%)
	Arterial catheter	1 (2.8%)
	Others	3 (8.3%)
**Antibiotic resistance**	Penicillin G	36 (100%)
	Oxacillin	30 (83.3%)
	Gentamicin	21 (58.3%)
	Erythromycin	23 (60.5%)
	Pristinamycin	0 (0%)
	Ofloxacin	23 (60.5%)
	Fusidic Acid	25 (69.4%)
	Rifampicin	5 (14%)
	Vancomycin	0 (0%)
	Teicoplanin	0 (0%)
	Linezolid	1 (2.8%)
	Trimethoprim/Sulfamethoxazole	18 (50%)

Table S2. Co-resistance of the *S. epidermidis* obtained after 15 days antibiotic exposure determined by MICs and interpreted following EUCAST recommendations

	Vancomycin MIC (mg/L)	Daptomycin MIC (mg/L)	Linezolid MIC (mg/L)	Ceftobiprole MIC (mg/L)
**5V**	4 (R)*	**4 (R)**	2 (S)	0.25 (S)
**26V**	4 (R)	**4 (R)**	2 (S)	0.5 (S)
**82V**	4 (R)	**4 (R)**	2 (S)	1 (S)
**97V**	4 (R)	**2 (R)**	2 (S)	1 (S)
**105V**	4 (R)	**4 (R)**	2 (S)	1 (S)
**82D**	**4 (R)**	2 (R)	2 (S)	1 (S)
**97D**	2 (S)	2 (R)	2 (S)	2 (S)
**105D**	2 (S)	2 (R)	2 (S)	2 (S)
**82C**	2 (S)	1 (S)	2 (S)	4 (R)
**105C**	2 (S)	1 (S)	2 (S)	4 (R)

*R, resistance; S, susceptible; In bold, coresistance generated with other antibiotics exposure

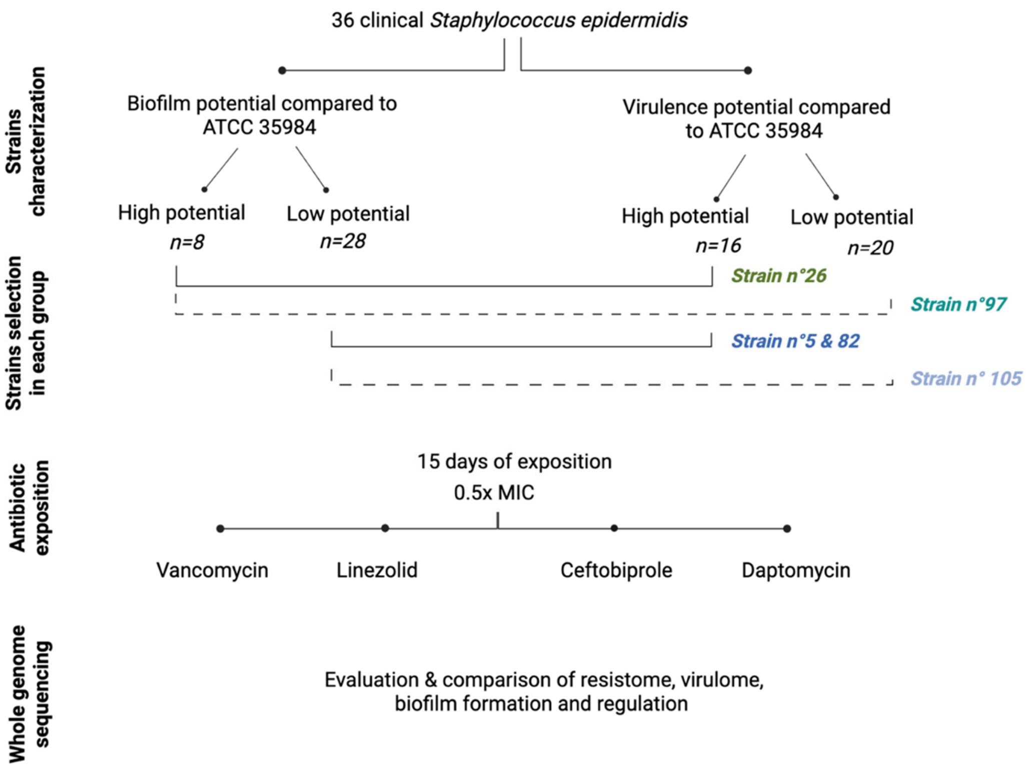

Figure S1. Protocol followed in this study to evaluate the impact of antibiotics exposure at sub-inhibitory MICs on *S. epidermidis* isolated from catheter-related bacteremia.
